# Regulation of Type 2 Immunity in Myocardial Infarction

**DOI:** 10.3389/fimmu.2019.00062

**Published:** 2019-01-29

**Authors:** Jun-Yan Xu, Yu-Yan Xiong, Xiao-Tong Lu, Yue-Jin Yang

**Affiliations:** ^1^State Key Laboratory of Cardiovascular Disease, Department of Cardiology, Fuwai Hospital, National Center for Cardiovascular Diseases, Chinese Academy of Medical Science and Peking Union Medical College, Beijing, China; ^2^National Cancer Center/National Clinical Research Center for Cancer/Cancer Hospital, Chinese Academy of Medical Sciences and Peking Union Medical College, Beijing, China

**Keywords:** myocardial infarction, type 2 immunity, interleukin, M2 macrophages, mast cells, eosinophils, immune modulation

## Abstract

Type 2 immunity participates in the pathogeneses of helminth infection and allergic diseases. Emerging evidence indicates that the components of type 2 immunity are also involved in maintaining metabolic hemostasis and facilitating the healing process after tissue injury. Numerous preclinical studies have suggested regulation of type 2 immunity-related cytokines, such as interleukin-4, -13, and -33, and cell types, such as M2 macrophages, mast cells, and eosinophils, affects cardiac functions after myocardial infarction (MI), providing new insights into the importance of immune modulation in the infarcted heart. This review provides an overview of the functions of these cytokines and cells in the setting of MI as well as their potential to predict the severity and prognosis of MI.

## Introduction

Type 2 immunity is characterized by the production of interleukin (IL)-4, IL-5, IL-9, IL-13, IL-25, IL-33, and thymic stromal lymphopoietin, as well as specific cell types including mast cells, eosinophils, basophils, alternatively activated M2 macrophages, type 2 innate lymphoid cells (ILC2), and T-helper (Th) 2 cells. It has mainly been considered to participate in the pathogeneses of helminth infection and allergic diseases. However, growing evidence suggests that these cell types and related cytokines are also involved in maintaining metabolic homeostasis and facilitating the healing process after tissue injury (1). Studies in experimental models and serum biomarker data from humans have proven the participation of type 2 immunity in the progression of myocardial infarction (MI). In this review, we will discuss several pivotal type 2 immunity-associated cytokines and cell types that modulate cardiac functions, following MI and their potential value as biomarkers of MI.

## Cytokines

Activation of innate immunity and extensive inflammation are the typical pathological features of MI. Accumulating evidence suggests type 2 cytokines are critical participants in tissue repair and regeneration owing to their ability to regulate the functions of nearby cells and immunomodulation. Moreover, they may serve as ideal biomarkers to predict the severity and clinical outcomes of MI.

### IL-4

IL-4 is an important Th2 cytokine with multiple biological functions, which mainly has an anti-inflammatory effect. Previous studies have demonstrated an association of elevated serum IL-4 with a reduced risk of cardiovascular diseases (2). Furthermore, the IL-4 level is lower in MI patients who later develop left ventricular dysfunction (3), indicating its cardioprotective properties.

One of the well-clarified mechanisms of IL-4 is in mediating myocardial repair via converting macrophages to the M2 phenotype. Administration of a long-acting IL-4 complex at 1 h after MI increases the proportion of cardiac M2 macrophages in both the infarct and border myocardium, along with increased tissue repair-related gene expression in M2 macrophages, and an improved cardiac structure (more connective tissue in the infarct zone) and functions. Further experiments suggested that IL-4 promotes fibrotic tissue formation via M2 macrophages rather than a direct interaction with cardiac fibroblasts. However, these effects are not observed when administrated at a late phase (7 or 28 days after MI), implying that IL-4 affects the early recruitment and polarization of M2 macrophages in the acute phase after MI (4). Similarly, injection of IL-4 plasmid DNA (carried by graphene oxide) around the border zone after coronary artery ligation largely reduces the number of inflammatory M1 macrophages, and polarizes macrophages to the reparative M2 phenotype in the mouse heart, leading to enhancement of cardiac functions (5).

IL-4 may also affect the functions of cardiac fibroblasts, thus participating in the profibrotic process directly. In the Ang II-induced hypertension model, wild-type (WT) mice exhibit higher cardiac fibrosis compared with *IL-4*^−/−^ mice, as indicated by the increase in the interstitial collagen fraction and mRNA levels of procollagen type-I α1 and procollagen type-III α1. *In vitro* experiments have demonstrated that IL-4 promotes the expression of procollagen type-I α1 and procollagen type-III α1 in mouse cardiac fibroblasts via binding to IL-4Rα, and consequently increasing the production of collagen (6). Treatment of anti-IL-4 neutralizing antibodies reduces both the number and proliferation of fibroblasts as well as infiltration of CD68^+^ macrophages (7). These findings suggest the sophisticated interaction between IL-4 and various cell types in the heart, which may lead to opposing outcomes under different pathological conditions.

### IL-13

IL-13 also polarizes macrophages to the M2 phenotype through binding to IL-4Rα and activating the subsequent signal transducers and activators of transcription (STAT) 6 signaling pathway (8). In a mouse model of MI, IL-13 significantly increases in the myocardium with a peak on day 3. Further experiments in *IL-13*^−/−^ mice suggested that IL-13 enhances cardiac functions by recruiting more monocytes/macrophages to the infarct and border area and inducing M2 macrophages. Interestingly, in contrast to the *IL-13*^−/−^ female mice, *IL-13*^−/−^ male mice exhibit a significant higher mortality and increased left ventricular dilation compared with WT mice after MI (9).

Recently, IL-13 was also found to induce mitosis of isolated cardiomyocytes when bound to IL-13Rα1. Through activation of the STAT3/periostin signaling pathway, IL-13 facilitates cardiac regeneration (10). Intraperitoneal administration of IL-13 significantly reduces the scar area and increases cardiomyocyte cell cycle activity/mitosis in a cardiomyocyte-specific *Gata4* knockout neonatal mouse after cryoinfarction (11). However, whether the salutary effects of IL-13 on the injured myocardium in the adult mouse model of MI are also partially related to its underlying regeneration property needs to be examined further.

### IL-33

IL-33, a member of the IL-1 family, has an important role in adaptive and innate immunities (12). After tissue injury, IL-33 released by the damaged endothelial or epithelial cells promotes immune cell recruitment and tissue repair (13, 14). In the heart, IL-33 is mainly released by cardiac fibroblasts responding to biomechanical stress (15). The cognate receptors of IL-33 have two isoforms: transmembrane ST2 (ST2L) and soluble ST2 (sST2) (16). The long form ST2L is expressed on various kinds of immune cells such as macrophages, mast cells, basophils, Th2 cells, regulatory T cells, and ILC2 (17–22). Gene ablation of *IL-33* or *ST2* has demonstrated that the IL-33/ST2 signaling pathway is crucial for reducing cardiac hypertrophy, ventricular chamber dilation, and cardiac fibrosis under mechanical stress (15, 23). However, the soluble form sST2, which serves as a decoy receptor, may impede the cardioprotective effects by neutralizing IL-33 (24). Accumulating evidence suggests that the IL-33/ST2 system has a profound effect on cardiac functions and potential value to predict the severity and prognosis of acute coronary syndrome (ACS).

In rats, IL-33 is elevated significantly within the first 12 weeks after MI. However, the mRNA level of sST2 shows a similar pattern to inflammatory and fibrosis markers with a peak at 1 week, suggesting that sST2 impairs the cardioprotective effects at an early stage post-MI (25). Preclinical studies have demonstrated that early pharmacological treatment targeting the IL-33/ST2 system promotes cardiac functions in MI rats. Through downregulating and upregulating gene expression of sST2 and IL-33, respectively, mineralocorticoid receptor antagonists reduce cardiac fibrosis and mitigate inflammation responses in the infarcted myocardium (26). Furthermore, β-blocker significantly decreases the infarct size and promote cardiac functions by reducing the sST2 level (27).

Further experiments showed that IL-33 reduces hypoxia-induced apoptosis of cardiomyocytes *in vitro* through suppressing caspase-3 activity and increasing anti-apoptotic protein expression (cellular inhibitor of apoptosis protein 1, X-linked inhibitor of apoptosis protein, survivin, B-cell lymphoma 2, and B-cell lymphoma-extra large). In a rat model of myocardial ischemia-reperfusion (IR) injury, subcutaneous injection of IL-33 significantly reduces the infarct size and myocardial fibrosis. The benefits of IL-33 on cardiac functions were then abolished by *ST2* gene deletion, indicating that IL-33 exerts cardioprotective effects through combination with the ST2 receptor (28). In the diabetic myocardium, a low level of IL-33 is associated with chronic activation of protein kinase (PK) CβII that increases the vulnerability of the myocardium to IR injury. Exogenous IL-33 supplementation reduces the phosphorylation of PKCβII, cardiomyocyte apoptosis, and infarct size after cardiac IR injury. In addition, anoxia/reoxygenation-induced apoptosis of high glucose preconditioned cardiomyocytes and activation of PKCβII are alleviated by IL-33 *in vitro* (29). IL-33 treatment also significantly suppresses proinflammatory cytokine and chemokine expression, including IL-1β, IL-6, IL-17, tumor necrosis factor-α (TNF-α), monocyte chemoattractant protein (MCP)-1, and interferon-γ (IFN-γ)-induced protein 10, and reduces macrophage infiltration after MI. These effects are mediated by inhibition of p38 mitogen-activated protein kinase and nuclear factor kappa-light-chain-enhancer of activated B cells pathways (30).

Human studies have demonstrated that the circulating levels of IL-33 and sST2 are associated with the severity of ACS patients, and may thus serve as potential biomarkers. The serum level of IL-33 is significantly lower in patients with ACS compared with stable angina pectoris patients and control individuals (31, 32). Similarly, another study showed that the circulating level of IL-33 is significantly lower in ACS patients than in patients with coronary artery disease (33). In contrast, sST2 is negatively correlated with the outcomes of MI patients. For MI patients, serum sST2 immediately elevated on day 1 after MI and correlates positively with peak creatine kinase and negatively with the left ventricular ejection fraction (LVEF) (34). In addition, a higher sST2 level is observed in patients with a larger infarct size, lower LVEF, transmural infarction, and microvascular obstruction (35). These findings indicate that the sST2 level well-reflects the severity of myocardial injury. Moreover, sST2 can predict both short term (36–39) and long term (39–43) cardiac adverse events and mortality in ACS patients.

## Cell Types

**Figure 1:**
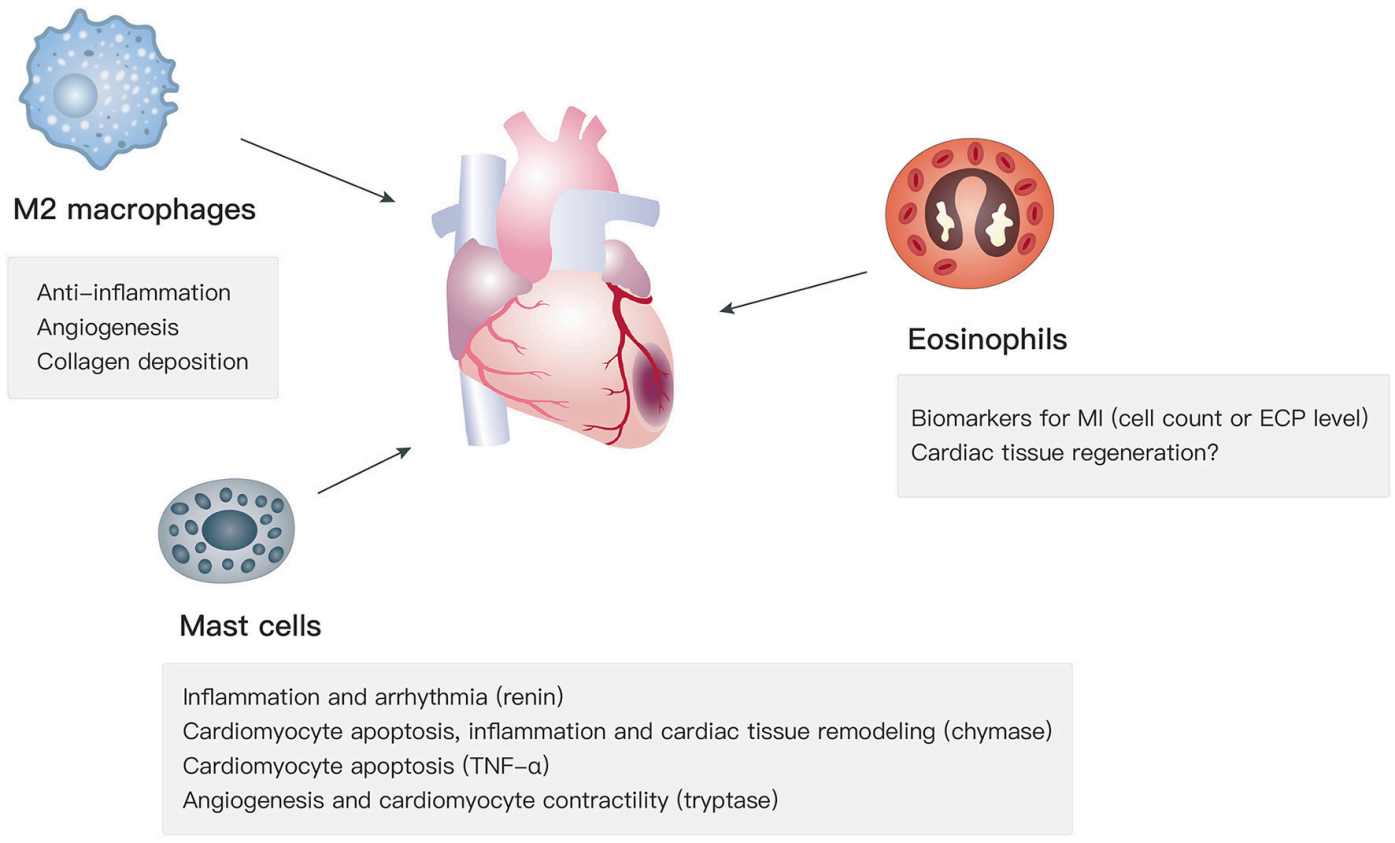
Functions of M2 macrophages, mast cells, and eosinophils in MI.

Apart from type 2 cytokines, the recruitment and activation of M2 macrophages, mast cells, and eosinophils, which are key type 2 immunity-related cell types, affect cardiac functions in the progression of MI via various mechanisms (Figure 1).

### M2 Macrophages

So far, two subsets of macrophages have been identified in the heart, according to their different origins: (1) resident cardiac macrophages derived from the yolk sac and fetal liver during embryonic development and (2) macrophages differentiated from circulating monocytes when they migrate into hearts (44, 45). Although there are less macrophages in the myocardium compared with cardiomyocytes, endothelial cells, fibroblasts, and smooth muscle cells (46), they are indispensable for both cardiac homeostasis and myocardial repair. Based on surface markers and gene expression profiles, macrophages are generally divided into classically activated M1 and alternatively activated M2 macrophages, although their phenotypes and functions might be more complex *in vivo* (47, 48). After MI, the injured myocardium sequentially mobilizes Ly-6C^high^ monocytes and Ly-6C^low^ monocytes via C-C chemokine receptor type 2 and CX3C chemokine receptor 1, respectively (49). Ly-6C^high^ monocytes differentiate into M1 macrophages, which dominate in the heart before day 3 post-MI and are responsible for degradation of the extracellular matrix and clearance of cellular debris; whereas Ly-6C^low^ monocytes differentiate into M2 macrophages that are the prominent subset during day 4–7 post-MI and mainly involved in the healing process (50). Accumulating evidence suggests that M2 macrophages participate in the resolution of inflammation and cardiac repair, which benefits cardiac functions after MI. In the next sections, we will summarize their subpopulations, biological functions, modulation methods, and polarization mechanisms.

#### Subpopulations

In response to different stimuli or pathological stresses, M2 macrophages polarize into distinctive phenotypes, namely M2a, M2b, and M2c (51, 52). M2a macrophages can be elicited by IL-4 or IL-13 with increased levels of CD206 (53) and arginase 1 (54), which support cell growth, collagen formation, and tissue repair by promoting the biosynthesis of polyamine and proline (55). Chemokines, such as C-C motif chemokine ligand (CCL) 2 (56), CCL17 (57), CCL22 (58), and CCL24 (59), are overexpressed in M2a macrophages, contributing to the recruitment of eosinophils, basophils, and Th2 cells. In addition, fibronectin, β IG-H3, and factor VIII subunit A are overexpressed in M2a macrophages, which are associated with extracellular matrix deposition and tissue remodeling (60, 61). However, the production of proinflammatory cytokines, including IL-1, IL-6, and TNF-α, is low in M2a macrophages (62), whereas the level of anti-inflammatory cytokines, including IL-10 and transforming growth factor-β (TGF-β), is high (63). M2b macrophages (elicited by immune complexes or Toll-like receptor ligands) are characterized by a low level of IL-12 and high level of IL-10. In contrast to elevated anti-inflammatory cytokines in M2a and M2c macrophages, M2b macrophages exhibit increased proinflammatory cytokines including IL-1β, IL-6, and TNF-α (64, 65). Another obvious distinction between M2b and M2a is that M2b cells have higher expression of sphingosine kinase 1 enzyme (66). They similarly regulate the recruitment of immune cells (eosinophils, Th2 cells, and regulatory T cells) by selective production of CCL1 (67). In terms of M2c macrophages, they are induced by IL-10, TGF-β, or glucocorticoid stimulation and express a high level of the surface marker CD163 (68) with decreased proinflammatory cytokines (IL-6, IL-12, and TNF-α) and proinflammatory mediators (inducible nitric oxide synthase and cyclooxygenase) (69). Previous studies have shown high quantities of matrix metalloproteinases (MMP)-7, MMP-8, MMP-9, and tissue inhibitor of metalloproteinase-1 in M2c macrophages, suggesting their potential to regulate fibrosis after MI (68, 70, 71). M2c macrophages also express high levels of chemokines CCL16 and CCL18 that attract naïve T cells and eosinophils (51).

#### Biological Functions: Anti-inflammation, Angiogenesis, and Collagen Deposition

**Figure 2:**
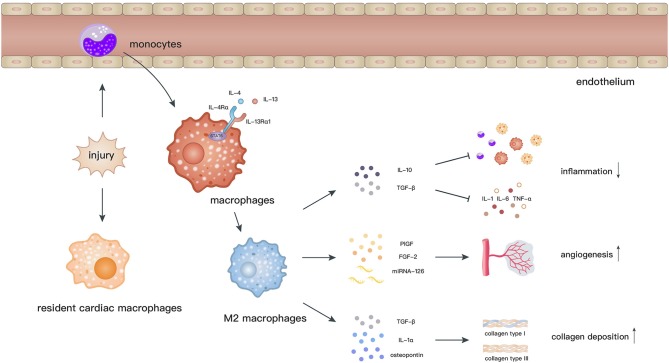
After myocardial ischemic attack, resident cardiac macrophages begin to develop apoptose by 2 h and circulating monocytes infiltrate into the injury site and differentiate into macrophages. Elicited by IL-4 and IL-13, macrophages polarize toward the M2 phenotype through activation of STAT6. M2 macrophages facilitate the recovery of cardiac functions via secretion of anti-inflammatory cytokines, promoting angiogenesis and collagen deposition.

Macrophages are related to the processes of initiation, maintenance, and resolution of the inflammatory response and myocardial repair after MI (72, 73). Cardiac resident macrophages begin to apoptose by 2 h and almost vanish within 24 h after MI. In contrast, a considerable number of monocytes are recruited into the myocardium and then differentiate into macrophages, which peak at day 6 after MI (74). M2 macrophages, which dominate the infiltration during day 4–7 post-MI, facilitate the recovery of cardiac functions via secretion of anti-inflammatory cytokines, neovascularization, and collagen deposition (72) (Figure 2).

##### Anti-inflammation

Previous studies have demonstrated that an exaggerated inflammatory response increases ventricular dilatation and cardiac dysfunction after MI (75), whereas attenuated inflammation suppresses scar formation (76), and increases the risk of cardiac rupture (77). Hence, timely resolution of inflammation is crucial for myocardial repair.

Owing to the ability to secrete pro/anti-inflammatory cytokines, macrophages are essential modulators of the inflammatory process after MI. In *apoE*^−/−^ atherosclerotic mice, prolonged presence of Ly-6C^high^ monocytes and higher proinflammatory gene expression in the infarcted myocardium hamper inflammation resolution and infarct healing (78), indicating the importance of timely infiltration by reparative M2 macrophages. Indeed, M2 macrophages restrict the expansion of inflammation through the release of anti-inflammatory cytokines including IL-10 and TGF-β. Further experiments demonstrated that IL-10 suppresses inflammation by restraining infiltration of inflammatory cells and the synthesis of inflammatory cytokines (IL-1β, IL-6, and TNF-α) *in vivo* (79). Early inhibition of TGF-β leads to increased infiltration of neutrophils and gene expression of IL-1β, TNF-α, and MCP-1, along with left ventricular dilation and decreased cardiac contractility, indicating that TGF-β protects the myocardium by regulating the inflammatory process (80).

##### Angiogenesis

Angiogenesis increases cardiac tissue perfusion, which makes it critical to salvage an infarcted myocardium. The beneficial effects of macrophages on cardiac functions are mediated partially by facilitating angiogenesis. Compared with WT mice, macrophage-deficient mice exhibit impaired angiogenesis and infarct healing (72). To further clarify the specific subtypes of macrophages that induce angiogenesis, circulating macrophages were depleted in the inflammatory phase (M1 macrophages) and healing phase (M2 macrophages), respectively. Consequently, there was a decline in quantity of microvascular α-actin^+^ smooth muscle cells and CD31^+^ endothelial cells in the infarcted myocardium when M2 macrophages were depleted (49). In addition, increased infiltration of M2 macrophages into myocardium after fibroblast growth factor (FGF)-2/hepatocyte growth factor administration is accompanied by enhanced angiogenesis (81). Simultaneously, M1, M2a, and M2c macrophages were injected subcutaneously into mice to determine their specific roles. In accordance with the above findings, compared with M1 macrophages, M2 macrophages had a higher angiogenic potential. When FGF-2 was neutralized in M2a or placental growth factor (PlGF) was blocked in M2c macrophages, angiogenesis and tube formation were reduced significantly, indicating that FGF signaling in M2a macrophages and PlGF signaling in M2c macrophages might be possible mechanisms of angiogenesis following MI (82). Apart from the release of angiogenic cytokines, M2 macrophages may regulate angiogenesis by transferring miRNAs. Angiogenic early outgrowth cells (EOCs), which are largely positive for M2 macrophage markers, were extracted from humans. Intramyocardial transplantation of EOCs from healthy donors into MI mice improved neovascularization in the infarct border zone and promoted cardiac repair. However, EOCs extracted from patients with chronic heart failure had loss of miRNA-126 and miRNA-130a and showed impaired cardiac neovascularization. Anti-miRNA-126 transfection decreased the angiogenic capacity of EOCs from healthy donors, whereas miRNA-126 mimic transfection increased the angiogenic capacity of EOCs from patients with chronic heart failure (83).

##### Collagen deposition

During the reparative phase after MI, collagen deposition in the infarcted myocardium stabilizes the damaged tissue and prevents infarct expansion and ventricular dysfunction. Depletion of macrophages decreases collagen deposition and wall thickness, increases left ventricular dilation, and leads to a high mortality after MI (72, 84). In contrast, injection of activated macrophages (73) or macrophage colony-stimulating factor (85) facilitates collagen deposition and myocardial repair.

M2 macrophage-depleted *Trib1*^−/−^ mice were used to identify the contribution of M2 macrophages to cardiac repair. *Trib1*^−/−^ mice exhibit decreased collagen fibril formation and more frequent cardiac rapture, whereas exogenous administration of IL-4, which promoted M2 macrophage polarization, increases the collagen volume in the infarct zone (86). Coculture with M2 macrophages isolated from the infarcted myocardium (86) or their secretome (87) enhances activation of cardiac fibroblasts *in vitro*. These effects might be ascribed to IL-1α and osteopontin, because gene expression of *Il1*α and *Spp1* is increased in M2 macrophages at 7 days after MI, and neutralization of IL-1α or osteopontin significantly reduces the fibroblast-myofibroblast transition when cocultured with M2 macrophages (86). Additionally, TGF-β released by M2 macrophages promotes synthesis of collagen type I and III (88, 89) through activation of Smad3 signaling in cardiac fibroblasts (90).

#### Modulation Methods and Polarization Mechanisms

Although numerous methods have been applied to promote the shift from M1 macrophages toward M2 macrophages after MI, the precise mechanisms of M1/M2 polarization have not been fully investigated in most studies (Table 1).

**Table 1:** Modulation methods and mechanisms of macrophage polarization.

**Modulation methods**	**Approaches**	**Animal strains**	**Pathological status**	**Polarization mechanisms**	**Biological effects**	**References**
**DRUG TREATMENT**						
BIO	Intraperitoneal	SD rats	MI	Not investigated	Cardiac fibrosis↓ Cardiac function↑	(91)
N-propargyl caffeamide	Intraperitoneal	SD rats	MI	Not investigated	Infarct size↓	(92)
DAPT	Intravenous	SD rats	MI	Not investigated	Arrhythmia↓ Sympathetic hyperinnervation↓	(93)
Pyridostigmine	Contained in water	Wistar rats	MI	Not investigated	Anti-oxidant enzyme activity↓ Cytokine production↓	(94)
Pyridostigmine	Contained in water	Wistar rats	MI	Not investigated	LV diastolic function↑ Parasympathetic modulation↑ Sympathetic modulation↑	(95)
Eplerenone	Intracerebroventricular	Wistar rats	MI	Not investigated	Cardiomyocyte apoptosis↓ LVEF↑	(96)
Atorvastatin	Intragastric	Wistar rats	MI	Not investigated	Arrhythmia↓ Sympathetic hyperinnervation↓	(97)
Dapagliflozin	Intragastric	Wistar rats	MI	STAT3 signaling pathway	Cardiac contractility and relaxation↑ Cardiac fibrosis↓ Oxidative and nitrosative stress↓	(98)
Nicorandil	Intragastric	Wistar rats	MI	RhoA/Rho-kinase signaling↓	Cardiac contractility and relaxation↑ Cardiac fibrosis↓	(99)
HGF and FGF-2 contained microparticle	Intramyocardial	Wistar rats	MI	Not investigated	Angiogenesis↑	(81)
Telmisartan	Intragastric	Zucker diabetic fatty rats	IR injury	Ubiquitin-proteasome system↓	Cardiac function↑ Infarct size↓	(100)
Sitagliptin + G-CSF	Contained in food and intraperitoneal, respectively	C57/BL6 mice	MI	Not investigated	Cardiomyocyte hypertrophy↓ LV dilatation↓	(101)
Niacin	Intragastric	C57BL/6 mice	MI	PGD_2_/DP1 axis↑	Cardiac function↑	(102)
Hydrogen sulfide	Intraperitoneal	C57BL/6 mice	MI	Lipolysis↑fatty acid oxidation↑	Cardiac function↑ Survival↑	(103)
IL-2/Anti-IL-2 immune complex	Intraperitoneal	C57BL/6 mice	MI	Not investigated	Cardiomyocyte apoptosis↓ Infarct size↓ LV function↑	(104)
Long-acting IL-4 complex	Intraperitoneal	C57BL/6 mice	MI	Not investigated	Angiogenesis↑ Cardiomyocyte hypertrophy↓ Connective tissue formation↑ Infarct size↓	(4)
Topiramate	Intraperitoneal	C57BL/6 mice	MI	Not investigated	Cardiac rupture↓ Collagen density↑ Infarct size↓ Survival↑	(105)
BAY 60-6583	Intravenous	C57BL/6 mice	IR injury	PI3K/PKB pathway↑	Infarct size↓ Inflammation↓	(106)
Suppressing IRF5 by siRNA	Intravenous	C57BL/6 mice	MI	IRF5	Infarct healing↑	(107)
IL-10	Subcutaneous	C57BL/6J mice	MI	Not investigated	ECM deposition↓ Inflammation↓ LV function↑	(87)
Ω-Alkynyl arachidonic acid	Intraperitoneal	C57BL/6N mice	MI	Regulating cross-talk between PKM2, HIF-1α and iNOS	CK-MB↓ Infarct size↓	(108)
CRMP2 siRNA	Intravenous	*ApoE^−/−^* mice	MI	IRF5↓	Cardiac fibrosis↓ Inflammation↓ LVEF↑ Scar size↓ Survival↑	(109)
Graphene oxide-carried IL-4 plasmid DNA	Intramyocardial	Balb/C mice	MI	Not investigated	Angiogenesis↑ Cardiac fibrosis↓ Inflammatory cell infiltration↓ LV function↑ Survival↑	(5)
Hemin formulated in designed lipid-based particles	Intravenous	Balb/C mice	MI	Not investigated	Angiogenesis↑ Infarct-related regional function↑ Scar tissue↓	(110)
Histone deacetylase inhibitor	Intraperitoneal	CD1 mice	MI	Not investigated	Angiogenesis↑ LV dilation↓ LVEF↑	(111)
FGF-9	Intramyocardial	Db/db diabetic mice	MI	Not investigated	Cardiac function↑ Infarct size↓ Inflammation↓	(112)
Ac-SDKP	Intraperitoneal	Mice	MI	Not investigated	Cardiac function↑ Collagen deposition↓	(113)
HBSP	Subcutaneous injection	Rabbits	MI	Not investigated	Coronary atherosclerosis↓	(114)
**GENE MODIFICATION**						
Depletion of Caveolin-1	Gene modification	*Cav1^−/−^* mice	MI	TGF-β/Smad2↑	Cardiac fibrosis↑ Inflammatory cell infiltration↑ Survival↓	(115)
Depletion of Lp-PLA_2_	Gene modification	*BmLp-PLA^−/−^* mice	MI	Not investigated	Angiogenesis↑ Collagen deposition↑ Infarct size↓ LVEF↑	(116)
Depletion of Wnt	Gene modification	*Cfms-icre;Wls^*fl*/*fl*^* mice	MI	Not investigated	Angiogenesis↑ Infarct-related regional function↑	(117)
Inhibition of PTP1B	Gene modification	*PTP1B^−/−^* mice	MI	Not investigated	Angiogenesis↑ LV Diastolic function↑ Myocardial perfusion↑	(118)
MIF deficiency	Gene modification	MIF deficient mice	MI	Not investigated	Cardiac remodeling↓ Cardiac rupture↓	(119)
Urokinase plasminogen activator overexpression	Gene modification	SR-uPA mice	MI	Not investigated	Cardiac fibrosis↑	(120)
**CELL TRANSPLANTATION AND TISSUE ENGINEERING**						
MSCs	Intramyocardial	SD rats	MI	Not investigated	Cardiac fibrosis↓ LVEF↑	(121)
MSCs	Intramyocardial	Macrophage depletion mice	MI	Not investigated	Infarct healing↑	(84)
BM-MSCs	Intravenous	NOD/SCID γ null mice	MI	IL-10 mediated	Cardiac function↑ Cardiac remodeling↓	(122)
FM-MSCs	Cell sheets	Lewis rats	MI	Not investigated	Angiogensis↑ Cardiac fibrosis↓ Cardiac function↑	(123)
Bone marrow transplantation	Intravenous	C57BL/6 mice	MI	Not investigated	Cardiac function↑ Cardiac remodeling↓ Survival↑ Wall thickness↑	(124)
Myocardial ECM patch	Sutured onto infarct area	Wistar rats	MI	Not investigated	Cardiac function↑	(125)
PHB patch	Patched on epicardial	Wistar rats	MI	Not investigated	Angiogenesis↑	(126)

*BIO, (2'Z,3'E)-6-Bromoindirubin-3'-oxime; DAPT, N-N-(3,5-difluorophenacetylL-alanyl)-S-phenylglycine-t-butyl ester; LV, left ventricular; HGF, hepatocyte growth factor; G-CSF, granulocyte-colony stimulating factor; PI3K/PKB, phosphatidylinositol 3-kinase/ protein kinase B; ECM, extracellular matrix; PKM2, pyruvate kinase isozymes M2; HIF, hypoxia-inducible factor; Inos, inducible nitric oxide synthase; CRMP2, collapsin response mediator protein 2; Ac-SDKP, N-acetyl-seryl-aspartyl-lysyl-proline; HBSP, helix B surface peptide; Smad, mothers against decapentaplegic homolog 2; Lp-PLA2, lipoprotein-associated phospholipase A2; PTP1B, protein tyrosine phosphatase 1B; MIF, macrophage migration inhibitory factor; SR-uPA, overexpression of urokinase plasminogen activator; MSCs, mesenchymal stem cells; FM-MSCs, fetal membrane-derived mesenchymal stem cells; BM-MSCs, bone marrow-derived mesenchymal stem cells; PHB, poly(3-hydroxybutyrate)*.

STAT proteins play an essential role in the immune response, inflammation, as well as cell growth and differentiation (127), and participate in various cardiovascular diseases (128, 129). It has been confirmed that IL-4 and IL-13 mediate macrophage polarization toward M2a macrophages depending on STAT6 signaling (130), whereas IFN-γ mediates macrophage polarization toward M1 macrophages depending on STAT1 signaling (131, 132). There is antagonism between STAT1 in M1 macrophages and STAT6 in M2 macrophages (133). Therefore, regulation of STAT1 and STAT6 axes is critical for the shift from M1 to M2 macrophages. Prostaglandin D_2_ (PGD_2_) participates in the resolution of inflammation (134) through binding to D prostanoid (DP1 and DP2) receptors (135). Macrophages express high levels of DP1 and DP2 (136), and activation of the DP1 receptor regulates macrophage infiltration and promotes inflammation resolution (137). In mice with macrophage-specific genetic deletion of DP1, macrophages are largely polarized to M1 phenotypes, leading to an extended inflammation period after MI with decreased myocardial repair. *In vitro* experiments showed that a DP1 receptor agonist inhibits Janus kinase 2/STAT1 phosphorylation by facilitating combination of the separated PKA regulatory IIα subunit and the transmembrane domain of IFN-γ receptor, which in turn induces STAT6 phosphorylation in macrophages (138). Similarly, another study confirmed that niacin activates the PGD_2_/DP1 axis to polarize macrophages toward the M2 subtype and promotes cardiac healing post-MI (102). In addition, STAT3 is widely recognized as the primary transcription factor modulating IL-10 signaling in macrophages, and activation of the STAT3 pathway is a potential mechanism for polarization toward M2c macrophages (139, 140). Dapagliflozin, a selective sodium-dependent glucose transporter inhibitor, acts as an antioxidant and enhances STAT3 activity during myocardial ischemia. Simultaneously, dapagliflozin preferentially activates M2c macrophages by increasing IL-10 expression and attenuating myofibroblast infiltration during post-infarction remodeling (98).

Apart from STAT, interferon regulatory factor (IRF) 5 has been identified as another transcription factor modulating M1 macrophage polarization (141). In IRF5-silenced mice, expression of a M1 macrophage marker decreases, and the resolution of inflammation and infarct healing are augmented (107). By silencing upstream gene expression of collapsin response mediator protein-2, the level of IRF-5 decreases, which is accompanied by an increase of M2 macrophages. Such an M1/M2 switch is reversed by overexpression of IRF5 (109). These studies provide novel gene modification strategies to modulate M2 macrophage polarization.

**Figure 3:**
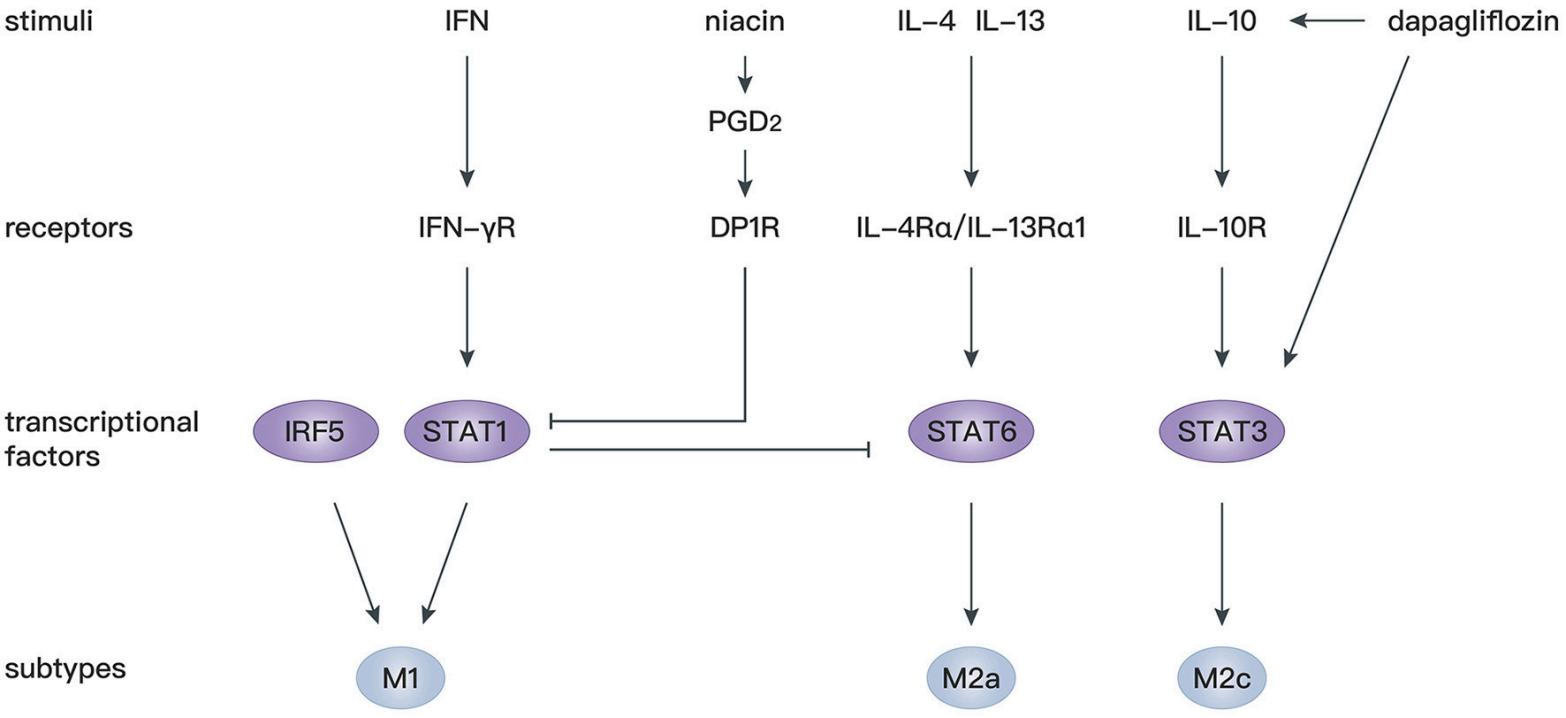
Mechanisms of macrophage polarization after MI.

Overall, targeting STAT and IRF signaling might be effective approaches to facilitate differentiation of macrophages toward the M2 phenotype, which is beneficial for cardiac repair after MI. More studies should be performed to investigate the precise mechanism of M2 polarization following MI (Figure 3).

### Mast Cells

**Figure 4:**
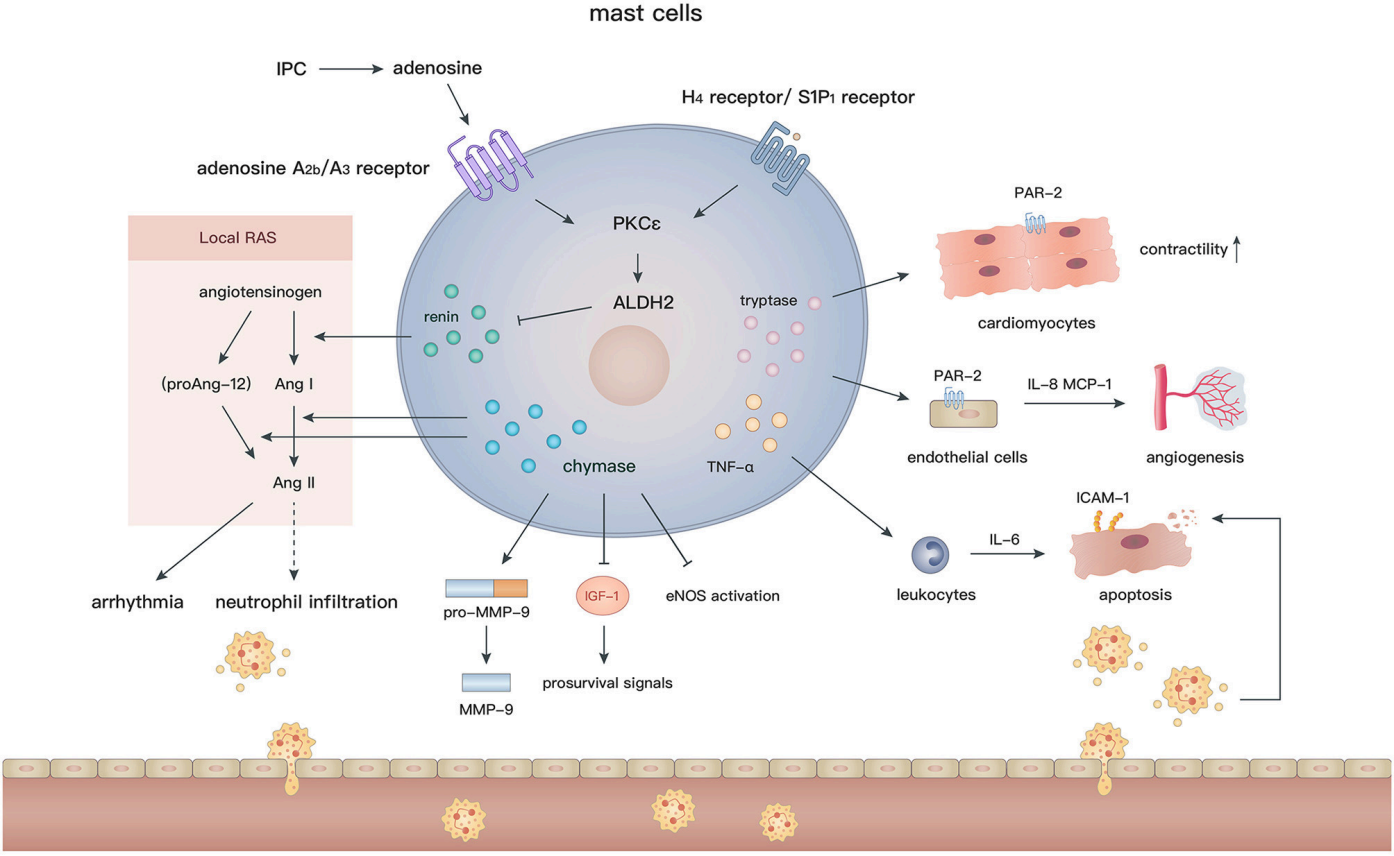
In the setting of MI, the diverse biological effects of mast cells are largely mediated by their granules containing renin, chymase, tryptase, and TNF-α. Degranulation of mast cells induces activation of local RAS, inflammatory cell recruitment, angiogenesis, and regulation of cardiomyocyte contractility and apoptosis.

Mast cells arise from hematopoietic pluripotent precursors in bone marrow and then mature in response to proper stimuli such as stem cell factor (c-kit ligand) and IL-3 (142). In contrast to the various phenotypes of macrophages, mast cells appear to be simpler and their effects are largely mediated by degranulation. With regard to their perivascular location and abundant bioactive granules, such as chymases, tryptases, histamine, renin, and cathepsins (143), mast cells are assumed to actively participate in cardiovascular diseases. Cardiac mast cells exist in both the hearts of humans (144) and animals (145, 146), and are essential to maintain aminopeptidase activity in the normal heart (147). In addition, many mast cells accumulate in the subepicardial layer of the infarct zone after MI (148, 149), indicating their involvement in the pathological process. Although numerous studies have been conducted to elucidate the role of mast cells after MI, the effects of mast cells on the ischemic or infarcted myocardium are still controversial (Figure 4).

#### Ischemia-Reperfusion (IR) Injury and Ischemic Preconditioning (IPC)

Although timely and efficient reperfusion is the most critical therapy for MI, it may also induce continuing necrosis of cardiomyocytes and exacerbate inflammation because of IR injury. IPC is an effective approach to reduce myocardial IR injury and improve cardiac functions (150). It has been demonstrated that mast cells contribute to the protective effects of IPC against IR injury in the small intestines (151) and cerebrum (152). However, in the setting of myocardial IR injury, current evidence indicates that mast cell granules are generally deleterious and might augment myocardial injury.

Earlier studies did not find any association between mast cells and IR injury or IPC after MI, because their numbers and granular content are not affected after IPC (153), and neither a mast cell stabilizer nor mast cell degranulating compound 48/80 influence the antiarrhythmic effects of IPC (154, 155). Nevertheless, mast cell peroxidase, which is a marker of mast cell degranulation, exhibits a remarkable increase in the coronary perfusate after IPC or compound 48/80 pretreatment, indicating the potential involvement of mast cell degranulation in IPC (156). Further experiments demonstrated that norepinephrine preconditioning reduces myocardial injury by promoting degranulation (157, 158), whereas adrenoceptor blocker (158) or mast cell stabilizer (159) treatments during IPC largely decrease the degranulation of mast cells, and thus mitigate the salutary effects of IPC. These findings imply that IPC facilitates discharge of toxic substances via premature mast cell degranulation and consequently alleviate detrimental effects during the following prolonged ischemia. Additionally, inhibition of mast cell degranulation by an adenosine A_2a_ receptor agonist (160) or relaxin (161, 162) at the reperfusion phase reduces the oxidative injury, infarct size, and ventricular arrhythmia in an IR model.

More recently, mast cells have been reported to be a crucial source of renin in the myocardium (163) and thus elicit post-IR arrhythmia by activating the local renin angiotensin (Ang) system (RAS) (164, 165). After IPC, the level of adenosine elevates rapidly in the myocardium (166). *Ex vivo* experiments showed that adenosine further activates the PKCε/aldehyde dehydrogenase type 2 (ALDH2) pathway in cardiac mast cells via combination with adenosine A_2b_/A_3_ receptors, in turn, reduces the local secretion of renin and biosynthesis of Ang II, which induces arrhythmia by modulating sympathetic nerve endings (167). In accordance with the above findings, activation of G_i_-coupled receptors, such as histamine-H_4_ and sphingosine-1-phosphate-S1P_1_ receptors on mast cells, also reduce the infarct size and the occurrence of arrhythmia through triggering the PKCε/ALDH2 pathway. In contrast, pharmacological inhibition of ALDH2 by glyceryl trinitrate treatment or gene modification (ALDH2^*^2 knock-in mice) abolishes the cardioprotective effects in IR models (168–170).

In addition to renin, IR injury can be caused by other granules in mast cells. Chymases effectively facilitate the conversion of Ang I (171, 172)/proAng-12 (173) (a proteolytic product of angiotensinogen) to Ang II, which may contribute to neutrophil infiltration via CXC chemokines (174) and cardiac tissue remodeling after IR injury. Interestingly, Ang II production is blocked by inhibition of chymases, but not Ang I-converting enzyme, suggesting that local chymase-induced Ang II production is independent from classic RAS activation. In fact, inhibition of chymases protects cardiomyocytes from apoptosis after IR injury by reducing the level of pro-MMP-9, cleaved MMP-9, and neutrophil infiltration, and increasing activation of endothelial nitric oxide synthase (175). Moreover, mouse mast cell protease 4 (a homolog of human chymase) depletion significantly reduces the late, but not early, infarct area and improves left ventricular functions by ameliorating insulin-like growth factor-1 degradation and activating subsequent prosurvival signals (176). In addition, under oxidative stress, TNF-α, which is released during mast cell degranulation, is recognized as a crucial substance that induces cardiomyocyte apoptosis after IR. TNF-α upregulates transcription of *IL-6* in recruited leukocytes and subsequent induction of intracellular adhesion molecule-1 in cardiomyocytes, which mediates neutrophil adherence to cardiomyocytes and neutrophil-mediated cardiomyocyte injury (177, 178). Mast cell stabilizers (ketotifen and cromoglycate) inhibit TNF-α secretion (179) and may attenuate myocardial injury after IR. These findings indicate that inhibition of mast cell degranulation or the release of specific granules may be a promising strategy to alleviate IR injury.

#### Cardiac Fibrosis

Studies have demonstrated the profibrotic properties of mast cells under various pathological conditions, such as atrial fibrillation (180), valvular heart disease (181, 182), and heart failure (183, 184). However, in MI, credible evidence is lacking for the correlation between mast cells and cardiac fibrosis, except for some indirect observations. Mast cell precursors are recruited in the area of collagen deposition at 2–3 days after reperfusion, which is mediated by macrophage-derived stem cell factor (185). In the chronic phase of MI, *in situ* hybridization demonstrated that plasminogen activator inhibitor-1, which induces tissue fibrosis by inhibiting MMPs, mainly lies in cardiomyocytes and perivascular mast cells around the infarction border zone (186). In a rat model of MI, inhibition of chymases significantly reduces the fibrotic area and mRNA levels of collagen I, collagen III, and TGF-β, which is important for the growth of fibroblasts (187). In addition, chymases facilitate the proliferation of fibroblasts in a dose-dependent manner *in vitro* (175). Additionally, bradykinin B_2_ receptor antagonist (Hoe140) administration reduces the number of myofibroblasts and attenuates interstitial fibrosis post-MI, in accordance with the reduction in mast cell infiltration (188). More studies are needed to ascertain the functions of mast cells in cardiac fibrosis and their underlying mechanisms in MI.

#### Protective Properties

Despite the long-held view that mast cells and their degranulation are detrimental to myocardial repair, studies continue to uncover their favorable effects. Clinical studies have shown that a high level of baseline serum immunoglobulin E (>200 IU/ml) is associated with less cardiac arrest or cardiogenic shock events in MI patients. It was speculated that immunoglobulin E facilitates mast cell infiltration and degranulation in the ischemic myocardium and thus improves the prognosis (189). Indeed, in a canine model of myocardial IR injury, mast cells accumulate along the cardiac vasculature for 4 weeks or longer and exhibit a defect in granular content (tryptases and chymases). *In vitro* experiments demonstrated that mast cell tryptases upregulate the expression of angiogenic cytokines by endothelial cells, including IL-8 and MCP-1, which might be mediated by protease-activated receptor 2 (PAR2) activation (149). In addition, mast cell-deficient rats (c-kit deficiency) exhibit a decreased coronary microvessel density around the infarct zone, a larger infarct core, and poorer left ventricular functions compared with WT rats (190). Hence, the infiltration of mast cells might promote the angiogenic activity of cardiac endothelial cells and subsequent healing process in the infarcted myocardium via tryptase secretion. However, c-kit deficiency affects the functions of mast cells as well as other immune cells. Models of specific depletion of tryptases, such as *Mcpt6*^−/−^ mice (191), are necessary to verify the effects of tryptases *in vivo*. Recently, a more reliable c-kit-independent mast cell-deficient (*Cpa3*^*cre*/+^) mouse was used to investigate the role of mast cells. Similarly, a large amount of mast cell progenitors, which mainly originated from white adipose tissue, were aggregated in the heart and differentiated into mature mast cells after MI. Although no differences were found in the capillary density, collagen deposition and the infarct size between *Cpa3*^*cre*/+^ and WT mice, it demonstrated that mast cell-derived tryptases inhibit PKA activation and subsequent troponin I and myosin-binding protein C phosphorylation by promoting PAR-2 activation and, in turn, increase the Ca^+^ sensitivity and contractility of cardiomyocytes (192).

The underlying cardioprotective abilities of mast cells have also been illustrated by direct transplantation (mast cells or their granular components). Mast cell granules (MCGs) obtained by collecting a cell suspension after compound 48/80 stimulation has been proven to be therapeutic in MI. Early MCG injection at the infarct site augments myocardial angiogenesis and reduces cardiomyocyte apoptosis. Treatment with MCGs enhances endothelial cell migration, tube formation, and hypoxic resistance of cardiomyocytes *in vitro* (193). In addition, intracoronary functional mast cell implantation promotes cardiac fibroblast-to-myofibroblast conversion and angiogenesis compared with non-functional mast cells (*Kit*^*W*/*W*−*V*^ mouse-derived mast cells), thereby preserving cardiac functions. However, these effects cannot be sustained long term (194). In addition, mast cells enhance cardiac functions by supporting the growth of stem cells. Mast cells or MCGs (extracted by freeze-thaw cycles and filtration) promote the migration and proliferation, but not myogenic differentiation, of mesenchymal stem cells (MSCs) via activation of the platelet-derived growth factor pathway in the early phase of MI. These effects may retain a sufficient number of MSCs for further myofibroblast differentiation in the healing phase (195).

Taken together, mast cell granules are very likely the main determinants in mediating beneficial effects after MI, including angiogenesis, cardiomyocyte contractility regulation, anti-apoptosis, hypoxia resistance, fibroblast-to-myofibroblast conversion, and the survival of stem cells. However, concerning the sophisticated composition of MCGs and different extraction methods, more studies are required to identify the key regulatory factors in their granules and to address the mechanisms using specific animal models.

### Eosinophils

Eosinophils differentiate from multipotent progenitors in bone marrow and are then released into peripheral blood. They contain various kinds of specific granular contents including eosinophil cationic protein (ECP), eosinophil peroxidase, major basic protein, eosinophil-derived neurotoxin, cytokines, growth factors, chemokines, and enzymes (196). As an indispensable component of type 2 immunity, eosinophils comprehensively interact with other immune cells and participate in the process of helminth infection and allergic diseases through degranulation activity. Recent data suggest that eosinophils are also involved in the progression of MI owing to their proinflammatory and prothrombotic properties.

#### Biomarkers for ACS

In MI patients, serum ECP elevates significantly during the initial 2–3 days, whereas the number of eosinophils in peripheral blood decreases, indicating that eosinophils probably infiltrate into the infarcted myocardium and participate in the acute inflammatory process after MI (197). The activation and degranulation of eosinophils in the infarcted myocardium may affect the structure of heart and lead to cardiac rupture (198).

Many studies have investigated the relationship between eosinophils or ECP and clinical outcomes of MI patients. Patients with a higher eosinophil-to-leukocyte ratio at 24 h after admission have significantly higher occurrence of major adverse cardiovascular events (199). Similarly, baseline ECP levels before stent implantation are higher in patients who suffer major adverse cardiac events such as cardiac death, recurrent MI, and clinically driven target lesion revascularization (200, 201). However, it was also reported that a high level of eosinophils (blood samples collected within 72 h after admission) is associated with a lower 1-year risk of death after multivariate adjustment (202). In addition, severe ACS patients have lower blood eosinophils compared with less severe ACS patients (203, 204). The inconsistent results of the relationship between eosinophil numbers and clinical outcomes of MI patients may due to the timing of blood sample collection or different patient cohorts.

By analyzing thrombus aspiration samples during emergency coronary angiography, eosinophils were found to be largely contained in the coronary thrombus of ACS patients and associated with a larger thrombus area, indicated that eosinophils caused the occurrence of MI by facilitating thrombus growth in the coronary artery (204, 205). In accordance with the above results, eosinophil degranulation, ECP levels, and the thrombus score were higher in ST-segment elevation MI patients with major adverse cardiac events at the 1-year follow-up (206).

#### Potential Mediator of Tissue Repair

Growing evidence has demonstrated that eosinophils also induce tissue repair. In a mouse model of cardiotoxin-induced tibialis anterior muscle injury, eosinophils largely aggregate in the injured site and activate the IL-4/IL-13 signaling pathway in fibro/adipogenic progenitors via secretion of IL-4. Consequently, the proliferation of fibro/adipogenic progenitors facilitate myogenesis. The regeneration ability is impaired in ΔdblGATA mice (unique loss of eosinophil lineage) (207). Similarly, eosinophils are recruited into the liver after hepatic injury and release IL-4 that directly promotes hepatocyte proliferation via blinding to IL-4Rα on these cells (208). However, studies concerning the role of eosinophils in the injured myocardium are lacking. It will be intriguing to further clarify the role of eosinophils in MI with regard to their specific abilities.

## Conclusion

Type 2 immunity-related cell types and cytokines participate in various physiological and pathological processes after MI. M2 macrophages inhibit the inflammatory response and promote angiogenesis and collagen deposition, thereby conferring benefits to the infarcted myocardium. Modulation of the macrophage polarization status is critical for myocardial repair. Although mast cells and their granules have been regarded as detrimental to myocardial healing, recent studies using more reliable mouse models have indicated that mast cell-derived tryptases actively regulate contractility of cardiomyocytes. Additionally, injection of MCGs preserves cardiac functions after MI by promoting angiogenesis, fibroblast-to-myofibroblast conversion, migration and proliferation of MSCs, and reducing cardiomyocyte apoptosis. In terms of eosinophils, the serum level of eosinophils and their granules, especially ECP, are closely related to the severity and clinical outcomes of ACS patients. Interestingly, two studies have revealed their underlying ability to activate intrinsic tissue repair of both muscular and hepatic injuries. However, these properties have not been tested in the setting of MI. Owing to the comprehensive interactions with immune and myocardial cells, type 2 cytokines have been proven to facilitate the recovery of cardiac functions after MI and serve as potential biomarkers to evaluate the severity and prognosis of MI. Nevertheless, the roles of basophils, ILC2, Th2 cells, and other type 2 cytokines in MI remain obscure. More studies are needed to further clarify the role of type 2 immunity in MI.

## Author Contributions

All authors made substantial contributions to the concept and interpretation of available evidence. J-YX, Y-YX, and X-TL drafted the manuscript and critically revised the manuscript for important intellectual content. All authors gave final approval of the manuscript for publication. All authors agree to be accountable for all aspects of the work and for ensuring that questions related to the accuracy or integrity of any part of the work are appropriately investigated and resolved.

### Conflict of Interest Statement

The authors declare that the research was conducted in the absence of any commercial or financial relationships that could be construed as a potential conflict of interest.
